# High-resolution analysis of the cytosolic Ca^2+^ events in β cell collectives in situ

**DOI:** 10.1152/ajpendo.00165.2022

**Published:** 2022-11-30

**Authors:** Sandra Postić, Srdjan Sarikas, Johannes Pfabe, Viljem Pohorec, Lidija Križančić Bombek, Nastja Sluga, Maša Skelin Klemen, Jurij Dolenšek, Dean Korošak, Andraž Stožer, Carmella Evans-Molina, James D. Johnson, Marjan Slak Rupnik

**Affiliations:** ^1^Center for physiology and pharmacology, Medical University of Vienna, Vienna, Austria; ^2^Institute of Physiology, Faculty of Medicine, University of Maribor, Maribor, Slovenia; ^3^Faculty of Civil Engineering, Transportation Engineering and Architecture, University of Maribor, Maribor, Slovenia; ^4^Center for Diabetes and Metabolic Diseases and the Herman B Wells Center for Pediatric Research, Indiana University School of Medicine, Indianapolis, Indiana; ^5^Richard L. Roudebush VA Medical Center, Indianapolis, Indiana; ^6^Diabetes Research Group, Life Sciences Institute, Department of Cellular and Physiological Sciences, University of British Columbia, Vancouver, British Columbia, Canada; ^7^Alma Mater Europaea—European Center Maribor, Maribor, Slovenia

**Keywords:** automated analysis, β cell, calcium dynamics, cell collective, pancreas tissue slices

## Abstract

The release of peptide hormones is predominantly regulated by a transient increase in cytosolic Ca^2+^ concentration ([Ca^2+^]_c_). To trigger exocytosis, Ca^2+^ ions enter the cytosol from intracellular Ca^2+^ stores or from the extracellular space. The molecular events of late stages of exocytosis, and their dependence on [Ca^2+^]_c_, were extensively described in isolated single cells from various endocrine glands. Notably, less work has been done on endocrine cells in situ to address the heterogeneity of [Ca^2+^]_c_ events contributing to a collective functional response of a gland. For this, β cell collectives in a pancreatic islet are particularly well suited as they are the smallest, experimentally manageable functional unit, where [Ca^2+^]_c_ dynamics can be simultaneously assessed on both cellular and collective level. Here, we measured [Ca^2+^]_c_ transients across all relevant timescales, from a subsecond to a minute time range, using high-resolution imaging with a low-affinity Ca^2+^ sensor. We quantified the recordings with a novel computational framework for automatic image segmentation and [Ca^2+^]_c_ event identification. Our results demonstrate that under physiological conditions the duration of [Ca^2+^]_c_ events is variable, and segregated into three reproducible modes, subsecond, second, and tens of seconds time range, and are a result of a progressive temporal summation of the shortest events. Using pharmacological tools we show that activation of intracellular Ca^2+^ receptors is both sufficient and necessary for glucose-dependent [Ca^2+^]_c_ oscillations in β cell collectives, and that a subset of [Ca^2+^]_c_ events could be triggered even in the absence of Ca^2+^ influx across the plasma membrane. In aggregate, our experimental and analytical platform was able to readily address the involvement of intracellular Ca^2+^ receptors in shaping the heterogeneity of [Ca^2+^]_c_ responses in collectives of endocrine cells in situ.

**NEW & NOTEWORTHY** Physiological glucose or ryanodine stimulation of β cell collectives generates a large number of [Ca^2+^]_c_ events, which can be rapidly assessed with our newly developed automatic image segmentation and [Ca^2+^]_c_ event identification pipeline. The event durations segregate into three reproducible modes produced by a progressive temporal summation. Using pharmacological tools, we show that activation of ryanodine intracellular Ca^2+^ receptors is both sufficient and necessary for glucose-dependent [Ca^2+^]_c_ oscillations in β cell collectives.

## Graphical abstract

**Figure:**
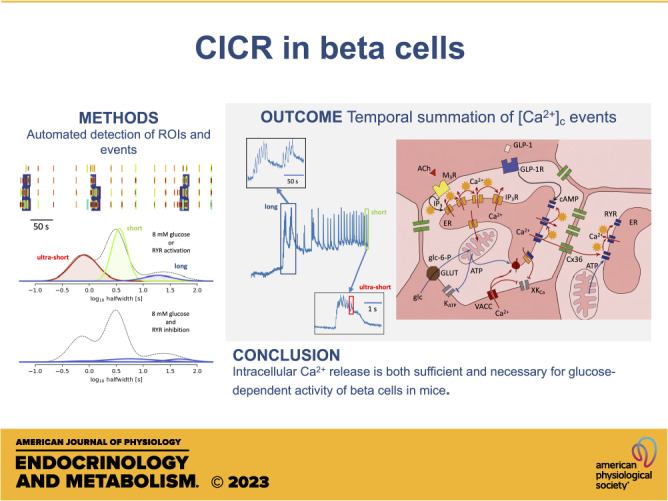

## INTRODUCTION

Beta cell exposure to high glucose stimulates insulin release. Glucose entry into the β cells through glucose transporters leads to a series of cellular events that trigger diffusion of Ca^2+^ ions into the cytosol. Resulting elevation in [Ca^2+^]_c_ ultimately produces a complex spatio-temporal pattern of [Ca^2+^]_c_ events, membrane potential changes, and exocytosis of insulin (1–3). Single-cell recording of electrophysiological parameters is accepted as the gold standard approach to follow the plasma membrane events in a β cell at a millisecond time scale (4, 5). Electrical bursts composed of subsecond spikes were measured to have a plateau frequency of a few per minute (4) at high physiological glucose, while measured [Ca^2+^]_c_ oscillations were often orders of magnitude slower, with a period in a time scale of minutes (6, 7). In the past, higher frequency and long-term recordings of [Ca^2+^]_c_ oscillations were limited due to the relatively weak fluorescence signal of the classical fluorophores, their high-affinity Ca^2+^ binding that yielded phase lags, slow sampling rates, and substantial bleaching (6–8).

There is growing evidence that intracellular Ca^2+^ release channels, namely the inositol (1,4,5)-trisphosphate (IP_3_) receptors (IP_3_R) and the ryanodine receptor (RYR), contribute to Ca^2+^-dependent insulin release in β cells (9–12). However, their exact role in physiological activation, activity, and deactivation of β cell is not clear (2). According to the current predominant view, these processes are primarily driven by plasma membrane voltage-activated Ca^2+^ channels (VACCs). RYRs are primarily located on endoplasmic reticulum (ER) membranes and can be activated by Ca^2+^ ions, ATP, and modulated by PKA (13). There are several factors that suggest that the actual role of IP_3_Rs and RYRs may have been underestimated. First, preincubation of β cells in 3 mM glucose empties Ca^2+^ from ER, putting intracellular receptors out of the game (14). Second, the maximal [Ca^2+^]_c_ concentration after glucose stimulation can reach values above 10 µM that cannot be picked up with high-affinity Ca^2+^ indicators, nor can they be achieved by a relatively low current density of VACCs on the plasma membrane (15). The [Ca^2+^]_c_ concentration achieved at the peak of the Ca^2+^ bursts is orders of magnitude above the resting values and slow oscillations around the baseline and is therefore physiologically of much higher significance. Third, Ca^2+^-induced Ca^2+^ release-like (CICR) phenomena in a broader sense could occur as cluster firing of fields of RYRs (or IP_3_Rs) (16). And fourth, the coexistence of both IP_3_Rs and RYRs that possess different Ca^2+^ activation and deactivation properties has been suggested as a basis for complex patterns of intracellular calcium regulation in other cell types (17). Furthermore, a reactive oxygen species (ROS)-dependent mechanism was described to stimulate RYRs to support glucose-dependent insulin release in male rats (18). Moreover, multiple studies suggest that disrupted activity of intracellular Ca^2+^ release channels may contribute to β cell dysfunction and glucose intolerance (19–21).

To assess the contribution of intracellular Ca^2+^ release to [Ca^2+^]_c_ changes requires a much higher temporally resolved imaging. Fortunately, the advancement in the synthesis of novel fluorescent Ca^2+^-sensing markers (22) solved most of the aforementioned technical issues enabling the recording of [Ca^2+^]_c_ with millisecond temporal and high spatial resolution simultaneously in many cells with a possibility of repeated stimulation of the same cells over prolonged periods. High data volumes generated in this manner demanded the development of an advanced data analysis, which is presented in this work. With these tools at hand, we conducted a series of experiments to revisit glucose-dependent β cell activation, activity, and deactivation, and with the help of pharmacological tools we reassessed the contribution of RYR intracellular Ca^2+^ release channels in these processes.

## MATERIALS AND METHODS

### Ethics Statement

We conducted the study in strict accordance with all national and European recommendations on care and handling experimental animals, and all efforts were made to minimize the suffering of animals. The Ministry of Education, Science and Research, Republic of Austria (No: 2020-0.488.800) and the administration of the Republic of Slovenia for Food Safety, Veterinary and Plant Protection (No: U34401-12/2015/3) approved the experimental protocol.

### Tissue Slice Preparation and Dye Loading

C57BL/6J mice, 8–20 wk of age, and of either sex (Jackson Laboratories), were kept on a 12:12-h light:dark schedule in individually ventilated cages (Allentown LLC) and used to prepare pancreatic tissue slices, as described previously (8, 23). In brief, after euthanizing the mice with CO_2_ and cervical dislocation, we accessed the abdominal cavity via laparotomy and distally clamped the common bile duct at the major duodenal papilla. Proximally, we injected the low-melting-point 1.9% agarose (Lonza) dissolved in extracellular solution [ECS, consisting of (in mM) 125 NaCl, 26 NaHCO_3_, 6 glucose, 6 lactic acid, 3 myo-inositol, 2.5 KCl, 2 Na-pyruvate, 2 CaCl_2_, 1.25 NaH_2_PO_4_, 1 MgCl_2_, 0.25 ascorbic acid] at 40°C into the common bile duct. Immediately after injection, we cooled the agarose-infused pancreas with ice-cold ECS and extracted it. We prepared tissue slices with a thickness of 140 µm with a vibratome (VT 1000 S, Leica) and collected them in HEPES-buffered ECS at RT [HEPES-buffered ECS, consisting of (in mM) 125 NaCl, 10 HEPES, 10 NaHCO_3_, 6 glucose, 6 lactic acid, 3 myo-inositol, 2.5 KCl, 2 Na-pyruvate, 2 CaCl_2_, 1.25 NaH_2_PO_4_, 1 MgCl_2_, 0.25 ascorbic acid; titrated to pH = 7.4 using 1 M NaOH]. For staining, we incubated the slices for 50 min at RT in the dye-loading solution (6 µM Calbryte 520, AAT Bioquest), 0.03% Pluronic F-127 (wt/vol), and 0.12% dimethylsulfoxide (vol/vol) dissolved in HEPES-buffered ECS. The Ca^2+^ fluorescent dyes from a Calbryte series are highly fluorescent and photostable, allowing long-term recording even in deeper layers of the tissue slice. The linear part of the Ca^2+^-binding curve for Calbryte 520 (*K*_D_ of 1.2 µM) captures the [Ca^2+^]_c_ changes in β cells better than the high-affinity sensors we routinely used in our imaging experiments before. All chemicals were obtained from Sigma-Aldrich (St. Louis, MO) unless otherwise specified.

### Stimulation Protocol and Cytosolic Calcium Imaging

We transferred individual pancreatic slices to a perifusion system containing 6 mM glucose in HEPES-buffered ECS at 34°C. We show representative traces for each experimental condition. Each of these conditions has been tested on at least three separate experimental days. Slices were exposed to a series of square-pulse-like stimulations characterized by exposure to 8 mM glucose for 25 min, followed by a washout with substimulatory 6 mM glucose concentration until all the activity switched off. Imaging was performed on standard confocal microscopes equipped with resonant scanners (Leica Microsystems TCS SP5 and SP8 or Nikon A1R) both upright and inverted with their respective sets of ×20 high numerical aperture (NA) objective lenses. Acquisition frequency was set to at least 20 Hz at 256 × 256 pixels, with pixels size close to 1 µm^2^ to allow for a precise quantification of [Ca^2+^]_c_ oscillations. Calbryte 520 was excited by a 488 nm argon laser (Leica SP5 and Nikon A1R) or 490 nm line of a white laser (Leica SP8). The emitted fluorescence was detected by HyD hybrid detector in the range of 500–700 nm using a photon counting mode (Leica) or GaAsP PMT detectors (Nikon).

### Analysis and Processing of Data

**Figure 1.**
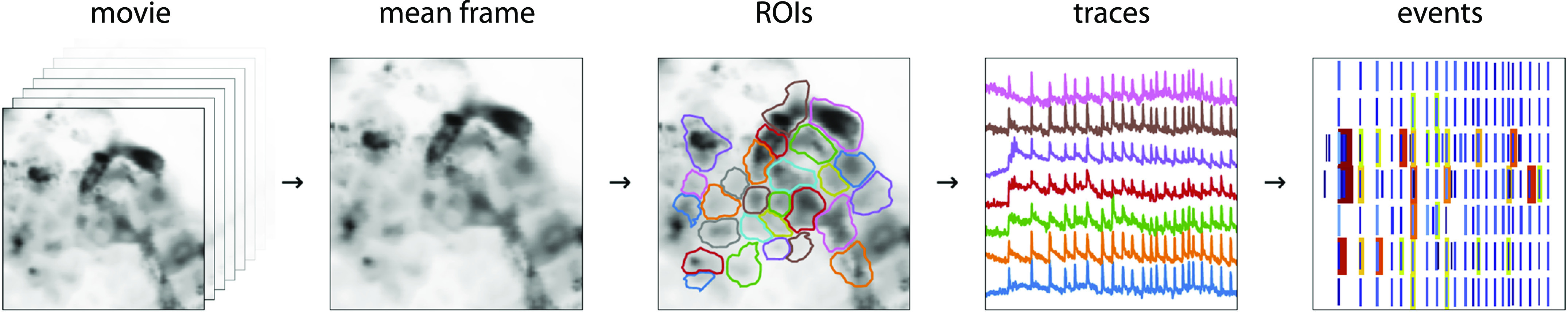
Processing pipeline to automatically detect regions of interest (ROIs) and [Ca^2+^]_c_ events at all time scales within an experiment. From a full movie, we calculated the mean (or other statistic) across all frames. We passed the mean image through a bandpass filter and define ROIs by detecting local peaks of light intensity. We then saved ROIs with all the important information (time traces, ROI coordinates, movie statistics, recording frequency, pixel size, etc.). Traces contained features at very different timescales—with different timescales presumably being important for different cell types. We collected them into separable events for analysis.

The general analysis pipeline was as follows. Experiments involving imaging of pancreatic slices typically focused on a single field of view showing up to hundreds of cells, in a recording of at least several, often dozens, gigabytes. Current tools that are widely used (e.g., ImageJ) rely on loading the recording, or its part, into memory, for viewing, analysis, and processing. It also requires laborious and long human engagement. We have developed a set of interdependent tools to automatize as much as possible the analysis pipeline (Fig. 1, Supplemental Material). Regions of interest (ROIs) were identified using a semi-automatic detection as follows. Recordings were stored as a three-dimensional (*T × X × Y*) numpy array (24). When the recording was stable, obtaining a mean image, or any other statistic over frame, was rather trivial. In case there was horizontal movement, it could be corrected for by aligning the frames to a template. For this we used the functionality present in CaImAn (25), except that high-frequency recordings needed to be rebinned to some moderate frequency (a few Hz), before correcting, to reduce the noise level. Once the translation offsets were obtained, we used them to correct the recording in original frequency. To define regions of interest, we blurred the representative image by a kernel of the size we expect cells to be, and at the scale double of that. The difference between these two images represents a bandpass filter of the original, where the local intensity variations were emphasized (Supplemental Fig. S7-1). We then passed through all pixels where the value of the filtered image was positive (or larger than a small positive threshold), and for each pixel we searched for a local peak in its vicinity. All the pixels that lead to the same local peak were then grouped into a single ROI. As we were mainly interested in islet cells, we chose the kernel size to approximately correspond to 10 µm, which is the characteristic length scale of the islet cells. If the pixel size is unknown, the choice of kernel is up to the person running the scripts. Representative image can be a mean over all frames or any other statistic. In addition, our code supports standard deviation, mean and standard deviation of the first derivative of the movie, and a “robust maximum” of the movie. As “robust maximum,” we defined a very high percentile of the set absolute values of a set, essentially a value close to its maximum, by default it is 10th largest. We avoid the maximum as a means to make analysis robust to outliers. This statistic is sensitive to cells that fire extremely rarely during a recording, so that the mean of those pixels is negligible. By default, we choose an average of the mean and high percentile as a representative image for bandpass filtering and ROI extraction.

Trace processing was conducted as follows. In an ideal detector, recording a time trace of a single pixel in the absence of any signal would consist of independent values of the number of photons detected during the dwell time. The values (*x*) are then distributed according to the Poisson distribution, with standard deviation (σ_1_) being equal to the square root of the mean µ_1_, ${\text{σ}}_{1}\;=\;\sqrt{{\text{µ}}_{1}}$. Transformation to standard score or *z*-score (Supplemental Fig. S7-2) $z\;=\;\frac{x-\text{µ}}{\text{σ}}\;$ is then performed that recasts the initial quantity *x* in the units of standard deviation from the expected mean.

A noisy trace in *z* spends 95% of the time between −2 and 2, and 99.7% between −3 and 3. Probability of *z* > 3 is very small *P* < 0.0013, which is why it is often considered a threshold value for pointing out the outliers. In general, the mean slow component needed to be inferred, typically by low-pass filtering. Throughout this project, we used cascaded second-order section (sos) filtering, implemented in scipy.signal module (25). The cut-off frequency *f*_cut_ of a filter determines the timescale of the events that can be detected in *z*-score.

Fast Fourier transform naturally distorts signals, but the inferred *z*-score can be used to correct for it. We constructed an iterative filtering algorithm, where at each iteration, we neglect the outlier values of the original trace, substitute them with the values of the slow component, and reapply the filter. At each iteration, the distortion is less prominent, increasing the *z*-score. In Supplemental Fig. S7-2, we show the result after 10 iterations, though we found three iterations as a more conservative choice, and a reasonable compromise between results and computing time.

All aforementioned refers also to a sum of pixel traces, but, crucially, not to their mean. A sum of two pixels *a* and *b* (*x* = *x_a_* + *x_b_*), with means µ_a_ and µ_b_, would have a standard deviation as expected $\sigma \;=\;\sqrt{\text{µ}}\;=\;\sqrt{{µ}_{\text{a}}+{µ}_{\text{b}}}$. But, if we were to consider the average $\overset{\text{¯}}{x}\;=\;x/2$, standard deviation would be $\sqrt{2}$ times smaller $\bar{\sigma }\;=\;\text{σ}/\sqrt{2}$. Therefore, when calculating *z*-score for a ROI trace, we always considered the sum, rather than the average trace of the underlying pixels. When we visualized traces, we show them averaged, only to keep the scales comparable. The same reasoning holds for rebinning a single trace, where the resulting trace, rebinned by a factor of *n*, had a standard deviation $\sqrt{n}$ times smaller than the original.

Experiments discussed in this manuscript were recorded on standard Leica SP5 or SP8, as well as NIKON A1R confocal microscopes. In cases where we used a Hybrid detector in the photon counting mode (Leica), we saw no significant departure from our assumption of Poisson distribution. Even with nonunit gain, the linear dependence between variance and mean remains, though the slope was different from 1 (Supplemental Fig. S7-3). Other types of detectors introduce additional sources of noise other than Poisson (e.g., thermal), but mostly they were still dominated by Poisson in our experience, at least as long as the values between neighboring pixels and frames were independent.

Traces contain features spanning orders of magnitude in time: from tens of milliseconds, to tens of minutes. We aimed to investigate how these events at different timescales and a connection between them and the islets’ environment interact. For this, we devised a two-step algorithm to identify intrinsic timescale of events and to minimize false positives (Supplemental Fig. S7-4). In the first step, we performed a sequential filtering of the traces at timescales starting from 0.5 s, and increasing by a factor of $\sqrt[{4}]{2},\;\text{τ}\;=\;(2^{-1},2^{-3/4},2^{-1/2},2^{-1/4},\ldots )$, until the timescale of the longest event of interest was achieved. At each timescale, we transformed the trace to *z*-score, and identify regions where *z* > 4 as candidate events.

Events were characterized by the start time (*t*_0_), its maximal height, and the width at the half of the height (half-width, δ*_t_*), which is our measurement of its duration. For simplicity, we defined end time as *t*_end_ = *t*_0_ + δ*_t_*, although events arguably last much longer after the intensity drops at half of the peak.

For an event to be considered real, it needed to be detected at multiple timescales, and will have started around the same time and will have approximately the same halfwidth. We specify a tolerance of 20% of the halfwidth as to whether two candidate events should be considered equal; if their start and end times were within 20% of the halfwidth, they were considered cognates, having arisen due to the same real event. For a set of cognates, we estimated the start and end time of the real underlying event as a median over the set. If the resulting estimate for the halfwidth is larger than 2 s, we required that a set consists of at least four candidate events (corresponding to event being detectable when filtered at timescales that differ at least twofold). For shorter events, we required only that an event is not unique to a single timescale.

We also neglected events that last less than three time-frames, as well as those too close to the beginning or end of the recording (within δ*t*/2), which we ascribed to artifacts from zero-padding for the filtering. We termed this procedure event distilling (Fig. 1, Supplemental Fig. S7-4).

## RESULTS

### Imaging of β Cell [Ca^2+^]_c_ in Pancreas Slices at Physiological Glucose Concentration

**Figure 2.**
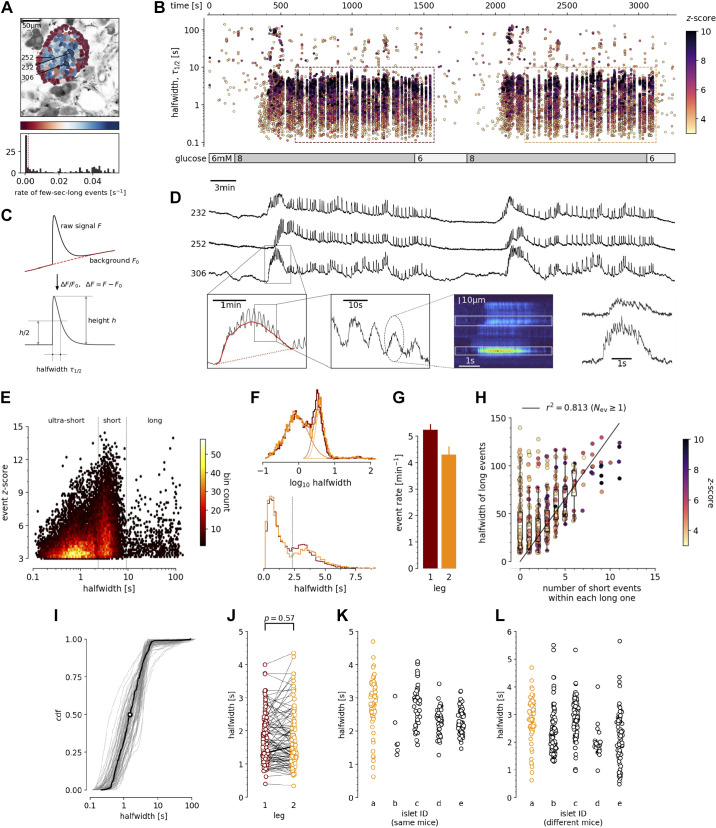
Quantification and analysis of [Ca^2+^]_c_ dynamics in physiological stimulatory glucose. *A*: regions of interest (ROIs) obtained by our segmentation algorithm. The color indicates the number of events identified in the ROI trace, upon a high-pass filtering at 0.2 Hz. We discarded ROIs with number of events below the threshold (red dashed line in the histogram in the *bottom*). Indicated are the ROI numbers whose filtered traces correlate best with the average trace for the whole islet; the correlation coefficients being 0.835, 0.818, and 0.807 (from top down in *D*). *B*: events’ halfwidth duration through time. Note the ranges of halfwidth duration occurring, the events’ synchronicity, and the phenotype’s reproducibility in sequential glucose stimulation. Color indicates the statistical significance in terms of *z*-score. Only events with a *z*-score higher than 3 were included. The stimulation protocol is indicated in the bar at the bottom of the pane. The plateau phases in both stimulations are indicated by a color coded rectangles. There is a prominent superposition of the short events on the plateau phases between the ROIs. *C*: an schematic of a transient event to describe the features of [Ca^2+^]_c_ events from background subtraction of the raw data, to height and halfwidth duration. Peak-point is the time of highest amplitude of an event. *D*, *top:* time courses from ROIs indicated in *A*, exposed to a double stimulation protocol, and rebinned to 2 Hz (recorded 20 Hz). The abscissa is shared with *B* and is indicated there. *Bottom*: illustration of the compounding nature of transients. The left-most panel is a closeup of the trace above to highlight the long transient in red. In the second closeup, we emphasize the structure within the long transient. The third panel shows data from a different experiment, in same conditions (8 mM glucose), recorded as a line scan. In the final panel, we plot the time course of the indicated spatial regions, to illustrate the structure of subsecond events. *E*: in a *z*-score vs. halfwidth density plot, the events are clearly separated into three groups, which we named ultra-short, short, and long, with a typical separation between the groups at 2 s and 8.5 s. The dominant time scale of the events had a halfwidth duration of 2–5 s. *F*: normalized Gaussian fit through the logarithmic distribution of halfwidth duration for events for both first and second stimulation as indicated in *B*. *G*: the rate of events for the dominant short [Ca^2+^]_c_ time scale for both stimulations indicated. *H*: evidence that the long events resulted from a progressive temporal summation of the (ultra-)short events. Some of the long events are less likely to contain substructure of short events, have lower *z*-scores, and contribute only little to the least square fit. *I*: cumulative distribution frequency (CDF) of the halfwidth duration of dominant short events during the plateau phase of the first stimulation for a randomly selected ROI. Thick black line indicates the median distribution. *J*: comparison between the halfwidth durations of events in individual ROIs during first and second glucose stimulation. *K*: comparison of halfwidth durations of events from all ROIs from different islets of the same mouse. *L*: comparison of halfwidth durations of events of all ROIs from islets of different mice.

Pancreas tissue slices offer the opportunity to study β cells in situ (Fig. 2; Supplemental Video S1) and closer to physiological conditions compared with isolated islets and dispersed islet cells (26). At substimulatory glucose concentrations (6 mM), β cells from adult mice displayed a stable resting [Ca^2+^]_c_, which can be seen during the prestimulatory and subsequent washout phases (Fig. 2, *B* and *D*). From a baseline of 6 mM, a physiological stimulatory glucose concentration (8 mM) after a typical delay of solution exchange and glucose metabolism, triggered a biphasic [Ca^2+^]_c_ response (Fig. 2, *B* and *D*). This biphasic response is composed of so-called initial increase in [Ca^2+^]_c_, previously termed the transient or asynchronous phase, followed by a prolonged plateau phase (Fig. 2, *B* and *D*) (8, 27). In both phases, we detected [Ca^2+^]_c_ events at different time scales, spanning from millisecond to a hundred of seconds range (Fig. 2, *B*, *D*–*F*, and *I*–*L*). These [Ca^2+^]_c_ events show several levels of temporal summation following a self-similarity pattern, as long events were a temporal summation of series of short events, a feature that could be indicative of CICR-like behavior (Fig. 2, *D* and *H*). In many aspects, the arrangement of short and long events reliably follows the electrical activity recorded in classical studies (4).

The initial transient phase is characterized by sizeable delays between the activation of individual β cell groups within an islet (28). In the initial phase, we recorded one or more large transient [Ca^2+^]_c_ events with a mean duration of tens of seconds (Fig. 2*D*), and with or without discernible superimposed shorter [Ca^2+^]_c_ events. The long transient events were also occasionally recorded outside the initial phase. In less than 30% of recorded islets or in a small fraction of the cells in a typical islet, long events were more prominent and were apparent in both initial as well as in plateau phase.

**Figure 3.**
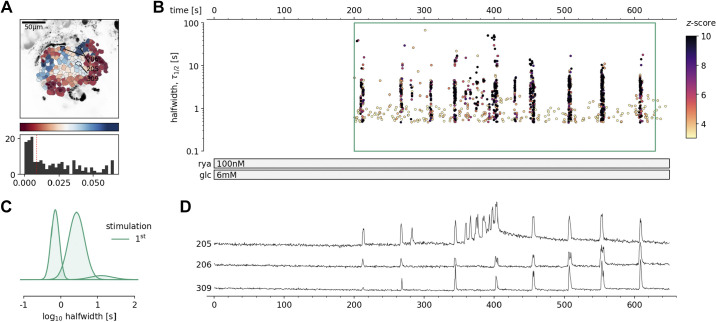
Pharmacological activation of intracellular ryanodine receptor (RYR) Ca^2+^ receptors in mouse β cells at subthreshold glucose concentration. *A*: regions of interest (ROIs) obtained by our segmentation algorithm. The color indicates the number of events identified in the ROI trace, upon a high-pass filtering at 0.2 Hz. We discarded ROIs with number of events below the threshold (red dashed line in the histogram in the *bottom*). Indicated are the ROI numbers whose filtered traces correlate best with the average trace for the whole islet. *B*: events’ halfwidth duration through time. Note the ranges of halfwidth duration occurring, the events’ synchronicity, and the phenotype’s reproducibility in ryanodine stimulation. Color indicates the statistical significance in terms of *z*-score. The stimulation protocol is indicated in the bar at the bottom of the pane. There is a prominent superposition of the short events on the plateau phases between the ROIs. *C*: normalized Gaussian fit through the logarithmic distribution of halfwidth duration during ryanodine stimulation, indicated temporal summation producing three discrete modes. *D*: time courses from ROIs indicated in *A*, exposed to a double stimulation protocol, and rebinned to 2 Hz (recorded at 20 Hz). The abscissa is shared with *B* and is indicated there.

Meanwhile, during the plateau phase we recorded mostly [Ca^2+^]_c_ events with a mean halfwidth duration of a few seconds (Fig. 2, *D*–*G*), occasionally exhibiting temporal summation into slowly rising and decaying long events (Fig. 2, *D* and *H*). These short events were a dominant time scale in the control conditions at 8 mM glucose in all experiments. The short [Ca^2+^]_c_ events within the plateau phase were commonly well synchronized among the β cells in an islet (Fig. 2, *B* and *D*). The short [Ca^2+^]_c_ events on the plateau phase were regenerative and could be stably recorded for hours at physiological glucose concentrations (29). Due to the regenerative nature of the short [Ca^2+^]_c_ events, we could design a multiple stimulation protocol on the same slice. The protocols were designed in a way that in the initial section we tested the responsiveness to the stimulation with 8 mM glucose, in the second section we tested the effect of a specific pharmacological treatment on stimulation with 8 mM glucose, and in the last section we applied another 8 mM glucose control stimulation. A detailed inspection of the short [Ca^2+^]_c_ events in the plateau phase showed that they were compound events composed of even shorter [Ca^2+^]_c_ events with a mean halfwidth duration below one second (Fig. 2*E*, Fig. 3*C*, and Fig. 6*C*). Similar ultra-short events were until now observed only as spikes on top of the bursts of the electrical activity, and were attributed to Ca^2+^ action potentials (30–32), mediated by the dominant contribution of L-type VACCs (33). Our approach offered insight into [Ca^2+^]_c_ events on times scales spanning several orders of magnitude (Fig. 2*E*), with temporal resolution comparable with that achieved with electrophysiological approach, and with an upgrade of simultaneously visualizing the activity of all islet cells in an optical plane permitting the interrogation of a collective behavior in situ (Fig. 2*A*).

The dynamic continuity of the time scales reflected in observed functional self-similarity resulted in a tri-modal distribution of halfwidth durations of the individual events that could be individually fitted with Gaussian function (Fig. 2*F*). The reproducibility of the individual modes between different experiments was high, suggesting relatively stable lengths of unitary and compound [Ca^2+^]_c_ events (Fig. 2*E*), and comparable degree of reproducibility and variability between the individual events on the plateau phase in the same ROI (Fig. 2*I*), within the same ROIs during sequential stimulation with glucose (Fig. 2*J*), within ROIs of different islet of the same mice (Fig. 2*K*), as well as among islets from different mice (Fig. 2*L*). However, the frequencies of the events of all time scales were typically not compared between different islets, since this parameter showed larger degree of variability, confirming previous reports (28). In addition, reporting shorter modes of [Ca^2+^]_c_ dynamics compared with classical approaches by using low-affinity indicators, we point out that these dominant short events reach at least an order of magnitude higher [Ca^2+^]_c_ and make a major contribution to Ca^2+^-dependent insulin release (34).

### Intracellular Ca^2+^ Channels Are Sufficient to Generate [Ca^2+^]_c_ Oscillations at Substimulatory Glucose

Next, we directly assessed the contribution of RYR intracellular Ca^2+^ channels, which in mouse β cells are predominantly RYR2 (21), in glucose-dependent [Ca^2+^]_c_ dynamics. We first tested the pharmacological RYR activation (Fig. 3, Supplemental Video 2). We showed that at subthreshold glucose, direct stimulation with stimulatory concentrations of ryanodine (100 nM) occasionally elicited regenerative Ca^2+^ events (Fig. 3). Predominantly short [Ca^2+^]_c_ events were triggered at stimulatory ryanodine concentration. Both short and long [Ca^2+^]_c_ events induced by ryanodine stimulation were a progressive temporal summation of the events in a subsecond range, and of the same duration as events observed under 8 mM glucose stimulation (Fig. 2). The onset of the activity varied between individual β cells in an islet (Fig. 3, *B* and *D*).

These results, together with the previously published data (35), suggest that pharmacological activation of both RYR and IP_3_ intracellular release channel could produce [Ca^2+^]_c_ events in β cells that would be regularly observed during the 8 mM glucose stimulation. Both stimulations generated compound [Ca^2+^]_c_ events through intermolecular CICR and presented two distinct kinetic components for intracellular release of Ca^2+^ and distinct regimes of intercellular coordination. Together, these data further confirm that a selective stimulation of Ca^2+^ release from the intracellular Ca^2+^ stores can contribute to [Ca^2+^]_c_ oscillations in β cells that are both coordinated (Fig. 3) and noncoordinated (35).

### Intracellular Ca^2+^ Channels Are Necessary for Glucose-Induced [Ca^2+^]_c_ Oscillations

**Figure 4.**
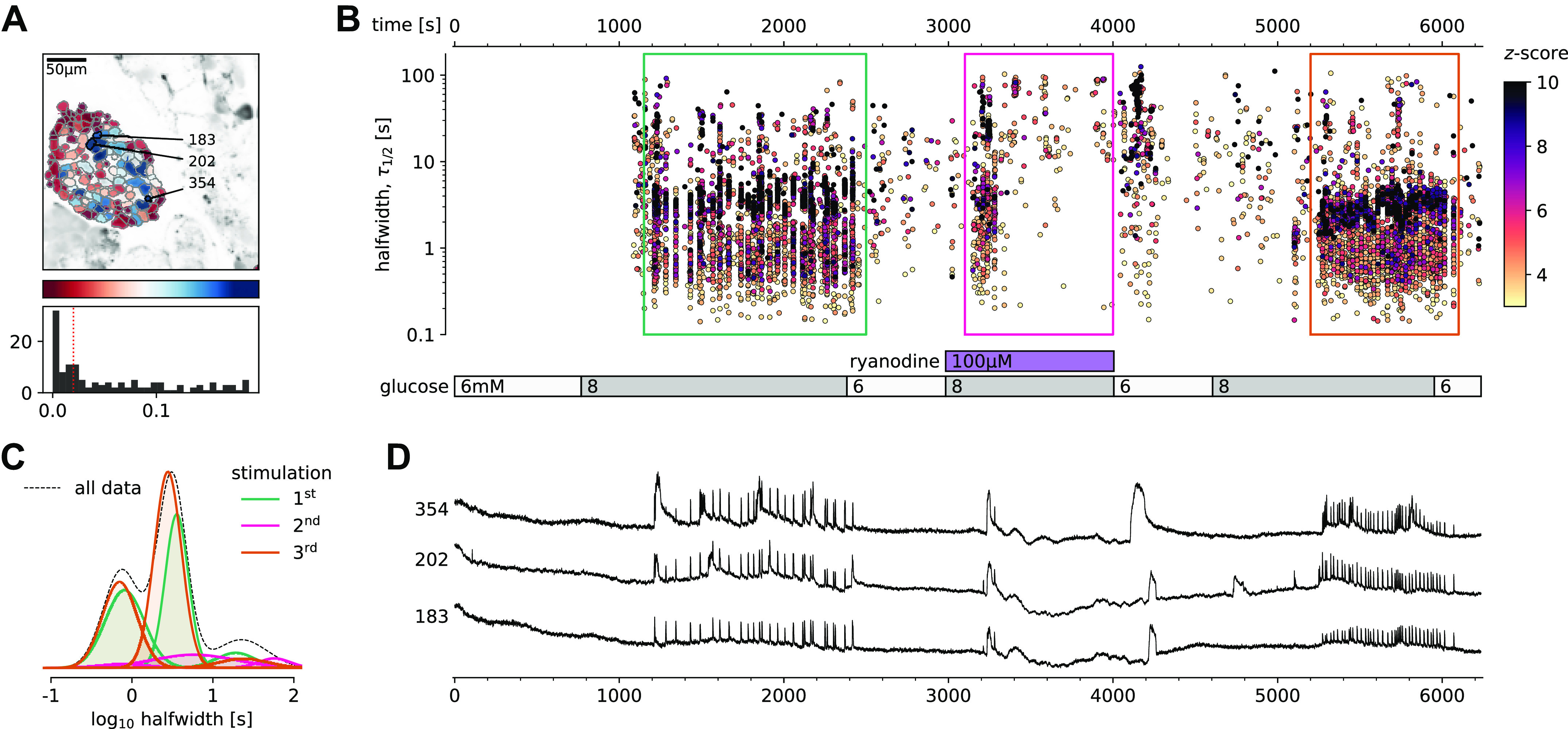
Pharmacological inhibition of intracellular ryanodine receptor (RYR) Ca^2+^ channels in mouse β cells selectively inhibits the plateau [Ca^2+^]_c_ oscillations. *A*: regions of interest (ROIs) obtained by our segmentation algorithm. The color indicates the number of events identified in the ROI trace, upon a high-pass filtering at 0.2 Hz. We discarded ROIs with number of events below the threshold (red dashed line in the histogram in the *bottom*). Indicated are the ROI numbers whose filtered traces correlate best with the average trace for the whole islet. *B*: events’ halfwidth duration through time for an islet exposed to a triple 8 mM glucose stimulation protocol. Inhibitory ryanodine (100 µM) was applied in the middle section of the protocol. The stimulation protocol is indicated in the bar at the bottom of the pane. There is a prominent superposition of the short events on the plateau phases between the ROIs. *C*: normalized Gaussian fit through the logarithmic distribution of halfwidth duration during control conditions and RYR inhibition, indicated temporal summation producing three discrete peaks. Note a complete absence of short events during the plateau phase of the second stimulation in the presence of high ryanodine. *D*: time courses from ROIs indicated in *A*, exposed to a triple stimulation protocol, and rebinned to 2 Hz (recorded at 20 Hz). The abscissa is shared with *B* and is indicated there. Note the reduced [Ca^2+^]_c_ level during the exposure to high ryanodine.

After demonstrating that [Ca^2+^]_c_ events can be evoked at glucose concentrations just below the glucose activation threshold with RYR activation, we used specific pharmacological tools to selectively block the activity of these intracellular channels and consequently determine the contribution of these receptors to the glucose-dependent stimulation of [Ca^2+^]_c_ events. We blocked RYR intracellular Ca^2+^ channels with an inhibitory ryanodine concentration (100 µM) (Fig. 4, Supplemental Video 3). High ryanodine selectively and completely inhibited the dominant time scale of events and its superimposed ultra-short events during the plateau phase, leaving initial long transient events intact (Fig. 4, *B* and *D*). Immediately after the washout of the high ryanodine, we observed prominent long [Ca^2+^]_c_ oscillations, which is consistent with stimulatory effects of the low ryanodine concentration (Fig. 4, *B–D*). The exposure to high ryanodine concentration was fully reversible and subsequent exposure to control 8 mM glucose stimulation resulted in a response comparable with the initial control stimulation (Fig. 4*B*).

### [Ca^2+^]_c_ Events Can Be Initiated and Maintained during L-Type VACC Block

**Figure 5.**
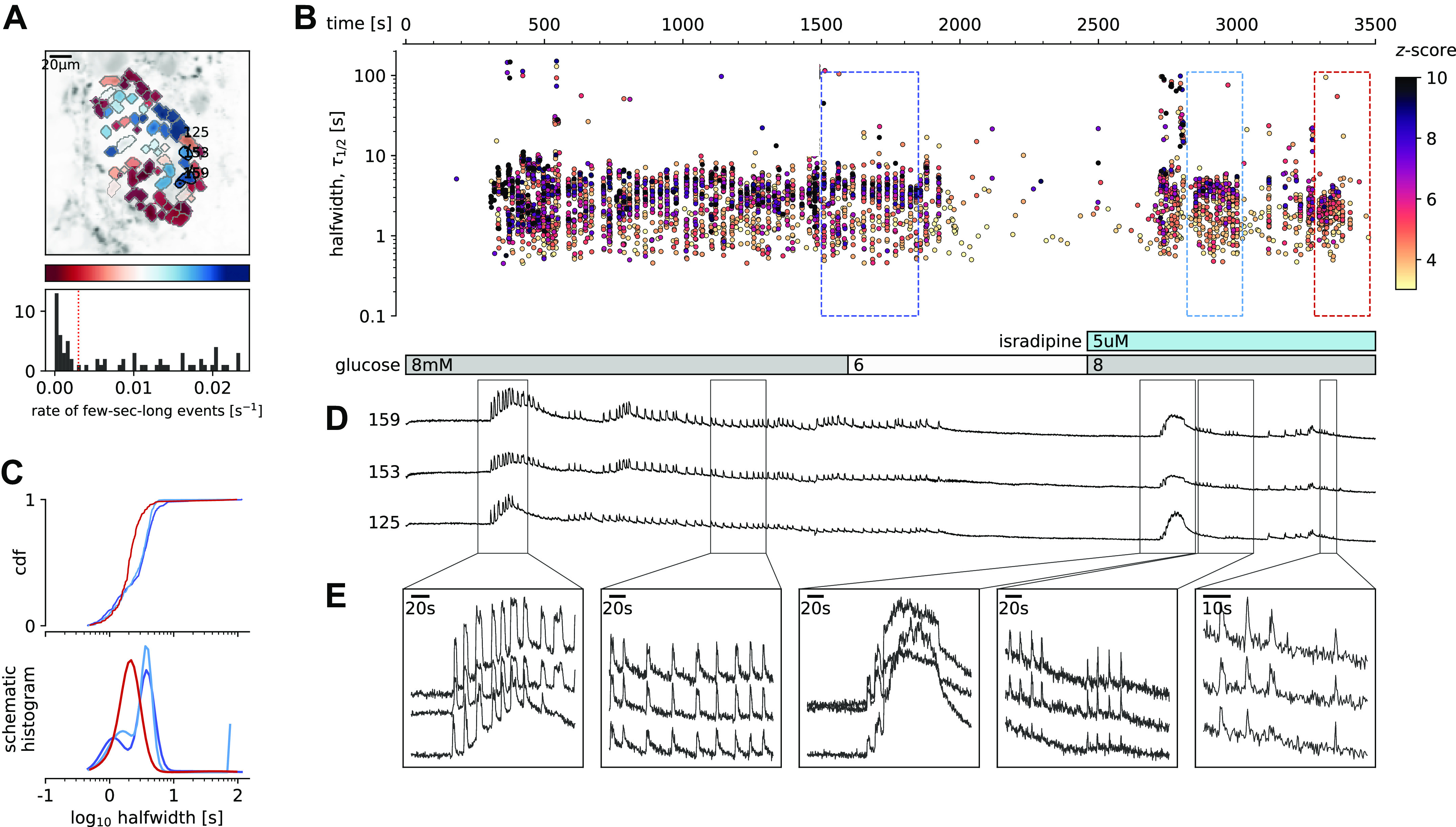
Glucose-dependent activation of β cells in the presence of inhibitory isradipine concentration to block voltage-activated Ca^2+^ channels (VACCs). *A*: regions of interest (ROIs) obtained by our segmentation algorithm. The color indicates the number of events identified in the ROI trace, upon a high-pass filtering at 0.2 Hz. We discarded ROIs with number of events below the threshold (red dashed line in the histogram in the *bottom*). Indicated are the ROI numbers whose filtered traces correlate best with the average trace for the whole islet. *B*: events’ halfwidth duration through time for an islet exposed to a double 8 mM glucose stimulation protocol. Saturating concentration of VACC blocker isradipine (5 µM) was applied in the second section of the protocol. The stimulation protocol is indicated in the bar at the bottom of the pane. There is a prominent superposition of the short events on the plateau phases between the ROIs. *C*, *top*: cumulative distribution frequency (CDF) of a mean halfwidth duration of events during the plateau phase of the both stimulations. *Bottom:* normalized Gaussian fit through the logarithmic distribution of halfwidth duration during control conditions and inhibition of VACCs. Note a shift toward shorter events during the late plateau phase in the presence of isradipine. *D*: time courses from ROIs indicated in *A*, exposed to a double stimulation protocol, and rebinned to 2 Hz (original frequency is 20 Hz). The abscissa is shared with *B* and is indicated there. *E*: expanded time traces from a representative ROI indicating (as indicated in *C*) the a long event from the initial transient phase, followed by a plateau phase in control, long event during initiation of the second stimulation, short events from early plateau phase, and further shortened events from a late plateau phase.

Our next step toward elucidating the molecular mechanisms of the aforementioned cytosolic Ca^2+^ events was to determine to what extent these multiple time scales of [Ca^2+^]_c_ events depended on the activity of L-type VACCs. Previous experiments performed on β cell-selective Cav1.2 Ca^2+^ channel null mice showed that, in the absence of the Ca^2+^ channel, [Ca^2+^]_c_ events were only moderately affected during the first minutes of the glucose stimulation, with a characteristic change in bursting pattern observed during acute stimulation (33). In the present study, we used the double stimulation protocol to test the effect of a saturating concentration of isradipine (5 µM), a specific L-type VACC blocker (Fig. 5, Supplemental Video 4). Consistent with the knockout mouse data, we observed a transient phase and initial plateau activity with a halfwidth duration with a median value of 3.1 s (*Q*1 1.8 s, *Q*3 4.6 s) during the first section and 3.4 s (*Q*1 2.1 s, *Q*3 4.3 s, *P* = 0.249) in the second section of the double protocol, where we co-applied isradipine. However, in the continuation of the plateau phase, the pattern of [Ca^2+^]_c_ events was characterized by shorter and smaller events with the median value of halfwidth duration of 2.4 s (*Q*1 1.9 s, *Q*3 3.1.s, *P* < 10^−15^) [Fig. 5*C* (red traces)]. Instead of CICR-like compound events of the dominant time scale, a reduced number of events with a mean halfwidth duration below 2 s remained that could reflect progressively lower probability of CICR after blockage of VACCs and a switch toward isolated ultra-short events (Fig. 5*E*). Within minutes after the change in the pattern of [Ca^2+^]_c_ events, the events disappeared completely.

Blocking L-type VACCs with isradipine during the plateau phase produced a similar change in the phenotype of [Ca^2+^]_c_ events, with progressively shorter and smaller events, before events subsided completely (Supplemental Fig. S4-1). A complete inhibition of the glucose-dependent [Ca^2+^]_c_ events was obtained when slices were exposed to 100 µM diazoxide before the onset of glucose dependence (Supplemental Fig. S4-2). At this concentration, diazoxide clamps the membrane potential close to the resting membrane potential, which is around the diffusion equilibrium potential for K^+^ (*E*_K_; −90 mV) (7, 36) and outside the range where VACCs could be activated (37). However, when we applied the same high diazoxide concentration on the plateau phase, as is shown in the Supplemental Fig. S4-3, similarly to isradipine, 100 µM diazoxide first changes the quality of the oscillations, before the activity stops completely in most of the cells or islets. One out of five islets did not switch off completely during 100 µM diazoxide. In line with the standard model, the washout of diazoxide in high glucose happened only very slowly, with a delay of 15 min after the start of diazoxide washout, as has been shown also by others (38). When β cells eventually activated, their oscillations were initially ultra-short and only with time developed into a dominant short phenotype.

Our experiments confirm a role of L-type VACCs for the regenerative glucose-dependent activity of β cells during the plateau phase (39). However, we also confirm previous observations that these channels play only part of the role during the first minutes of β cells activation, and that the plasma membrane depolarization plays an independent role in stimulation of intracellular Ca^2+^ release (40).

### [Ca^2+^]_c_ Oscillations in Low Extracellular Ca^2+^ Conditions

**Figure 6.**
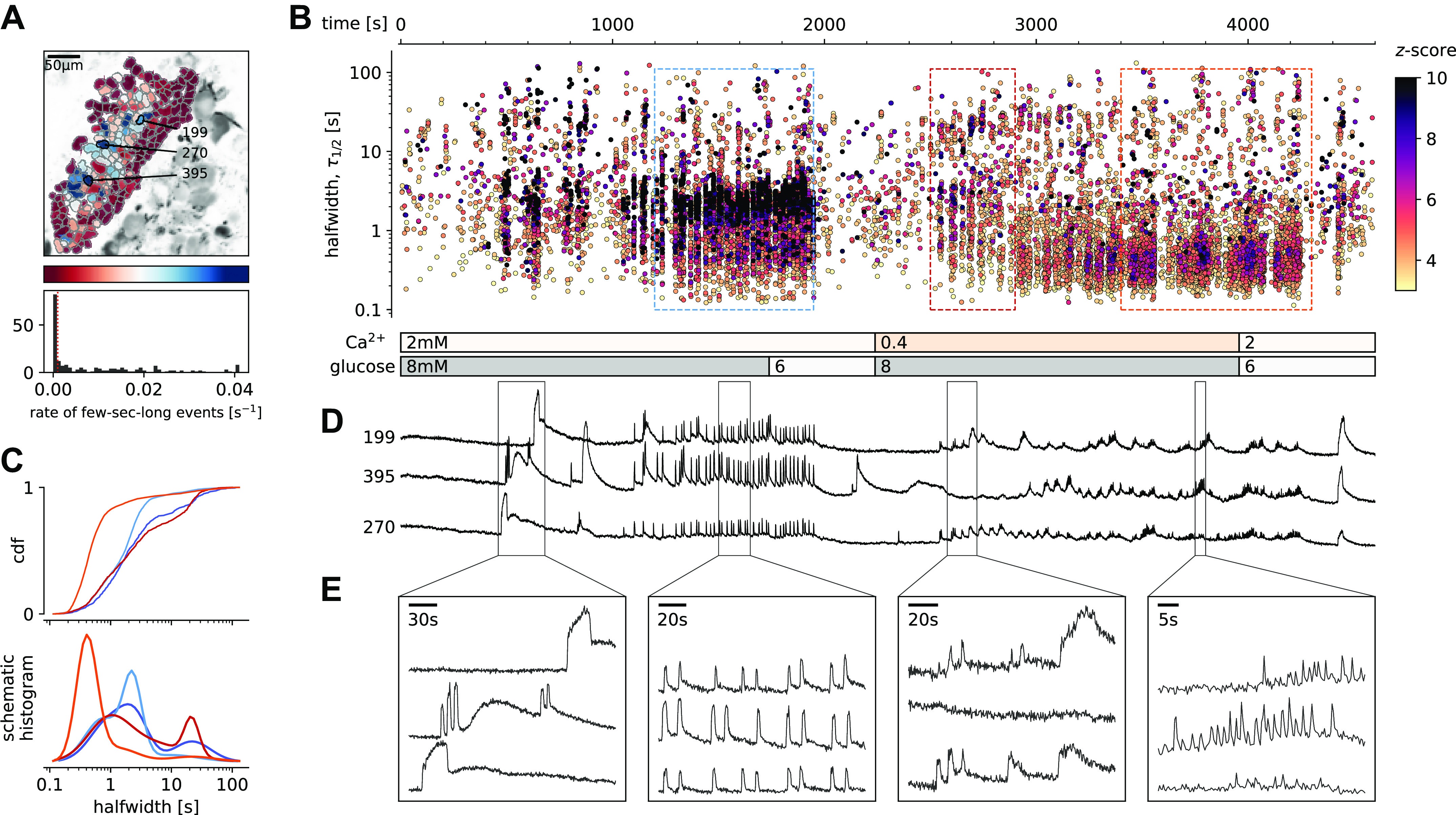
Glucose-dependent activation of β cells at subphysiological extracellular Ca^2+^ concentration. *A*: regions of interest (ROIs) obtained by our segmentation algorithm. The color indicates the number of events identified in the ROI trace, upon a high-pass filtering at 0.2 Hz. We discarded ROIs with number of events below the threshold (red dashed line in the histogram in the *bottom*). Indicated are the ROI numbers whose filtered traces correlate best with the average trace for the whole islet. *B*: events’ halfwidth duration through time for an islet exposed to a double 8 mM glucose stimulation protocol. Subphysiological extracellular Ca^2+^ level (400 µM) was applied in the second section of the protocol. The stimulation protocol is indicated in the bar at the bottom of the pane. There is a prominent superposition of the short events on the plateau phases between the ROIs. *C*, *top:* cumulative distribution frequency (CDF) of a mean halfwidth duration of events during the plateau phase of the both stimulations. Events from the initial transient phase are in dark colors, events from the plateau phase are in light color. *Bottom:* normalized Gaussian fit through the logarithmic distribution of halfwidth duration during control conditions and inhibition of voltage-activated Ca^2+^ channels (VACCs). Note a shift toward shorter events during the plateau phase in the conditions of subphysiological extracellular Ca^2+^ concentration. *D*: time courses from ROIs indicated in *A*, exposed to a double stimulation protocol, and rebinned to 2 Hz (recorded at 20 Hz). The abscissa is shared with *B* and is indicated there. *E*: expanded time traces from a representative ROI indicating (as indicated in *C*) the a long event from the initial transient phase, followed by a plateau phase in control, long event during initiation of the second stimulation, and short events from early plateau phase in low extracellular Ca^2+^ concentration.

To further test the potential role of intracellular Ca^2+^ channels on ER in the spatio-temporal regulation of [Ca^2+^]_c_ events, we used a classical approach with the reduction of extracellular Ca^2+^ concentration (40). Incubation of β cells with 0.4 mM extracellular Ca^2+^ uncovered two major phenomena (Fig. 6, Supplemental Video 5). First, β cells within the islets tended to functionally dissociate, losing coordinated and global intercellular Ca^2+^ events with a phenotype resembling the Cx36 ablated or pharmacologically blocked cell-cell electrical coupling (41, 42). Second, low extracellular Ca^2+^ also changed the pattern of [Ca^2+^]_c_ events (Fig. 6). The most prominent effect of low extracellular Ca^2+^ concentration was the complete absence of the short dominant time scale events (Fig. 6), where the median halfwidth duration of 2.2 s (*Q*1 1.6 s, *Q*3 3.0 s) from the control section, decomposed to ultra-short events with a median halfwidth duration of 0.6 s (*Q*1 0.5 s, *Q*3 1.7 s, *P* < 10^−200^) during the treatment section. In addition, the median amplitude of the events was significantly smaller in the section with low extracellular Ca^2+^ concentration. The decomposition of the dominant time scale short [Ca^2+^]_c_ events resulted in a series of smaller, but higher frequency events (Fig. 6, *B–E*), confirming previous electrophysiological measurements(7). The coherence of responses between the individual β cells within the islet was more localized in low extracellular Ca^2+^ concentration. Also long events were significantly longer in the low extracellular Ca^2+^ concentration with median halfwidth duration of 24 s (*Q*1 14 s, *Q*3 33 s) in comparison to 184 20 s (*Q*1 11 s, *Q*3 32 s, *P* < 0.0001) during the control section. Our results suggest that in the presence of sufficient extracellular Ca^2+^ concentration, the CICR-like [Ca^2+^]_c_ oscillations are likely to occur in glucose-stimulated β cells in situ and that these events decompose at subphysiologically low extracellular [Ca^2+^]. The spectrum of recorded patterns of β cell activities confirms previously described role of CICR through intracellular Ca^2+^ channels in glucose-dependent activity of these cells (43, 44).

The low extracellular Ca^2+^ concentration recordings provide further evidence regarding the existence of two distinct phases for the activation and activity of β cells. The initial phase during the activation of β cells in the fresh pancreas slice consisted of one or few long IP_3_-dependent [Ca^2+^]_c_ transients (some tens of seconds long) as reported earlier (35), followed by a plateau phase comprising of a series of short RYR-mediated events.

## DISCUSSION

Decades of electrophysiological experiments on pancreatic β cells have established a critical role for plasma membrane ion channels in controlling excitability, dynamics of cytosolic Ca^2+^ events, and insulin exocytosis (2, 30). In standard electrophysiology experiments, β cells are kept at 3 mM glucose where they are electrically silent, after which they are activated by an instantaneous increase to glucose levels well in excess of 10 mM (28, 42).

**Figure 7.**
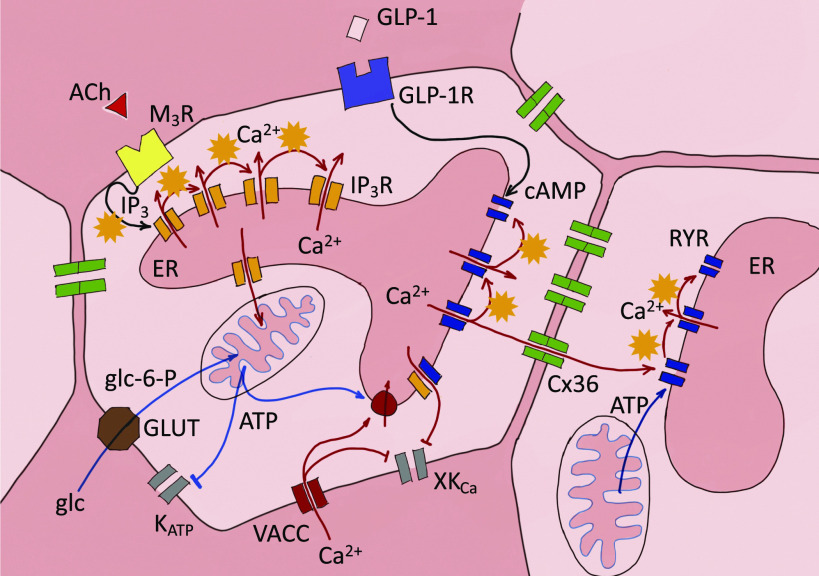
Proposed model of the role of intracellular ryanodine receptor (RYR) and inositol (1,4,5)-trisphosphate (IP_3_) Ca^2+^ release channels in activation and activity of the mouse β cell. The basic principle in different patterns of [Ca^2+^]_c_ oscillation is intermolecular Ca^2+^-induced Ca^2+^ release-like (CICR, orange stars). Physiological glucose stimulation activates both IP_3_R and RYR activity, which is followed by repetitive bursts of RYRs activity. The subcellular arrangement of RYRs enables intercellular communication through the Cx36 proteins and synchronized propagation of the Ca^2+^ events. ATP-dependent closure of K_ATP_ channels contributes to the synchronization. VACCs are critical for refilling of the internal stores. [Ca^2+^]_c_ changes are registered by different plasma membrane K^+^ channels (XKCa). GLP-1R stimulation of cAMP production and ATP from the mitochondrial metabolism can modulate the RYR activity. Ca^2+^ concentration in the extracellular space and in different compartments of the cell is color coded, with the lowest [Ca^2+^]_c_ in the cytosol.

In the current study, we combined fast confocal microscopy, a photostable and bright low affinity Ca^2+^ sensor, and tools of data sciences to obtain new insights into physiological activation of β cells within intact islets in fresh pancreas tissue slices. Specific pharmacological modulation of RYR in intact β cell collectives stimulated with 8 mM glucose and recorded with a unique spatio-temporal resolution demands an updated model of β cell activation and bursting activity. According to our model, multiple time scales of events are generated with a progressive temporal superposition of ultra-short events, which represent CICR of either IP_3_ (35) or RYR Ca^2+^ release channels (Fig. 7). Both intracellular channels can be directly stimulated by glucose. The ultra-short events represent unitary activity of intracellular Ca^2+^ release channels, which can engage in intermolecular CICR, and as described for other cell types, depend on sufficient Ca^2+^ ER load (16, 45).

Evidence for CICR in β cells involving both IP_3_ and RYR channels has been previously reported (46–48). When, however, the ER Ca^2+^ load decreases, CICR bursts switch to subsecond events produced by individual or more localized clusters of intracellular Ca^2+^ channels before events eventually stop. Decomposed CICR bursts, therefore, appear to oscillate at higher frequency, a phenomenon reported after ER emptying using thapsigargin (49), genetic ablation of L-type VACCs (33), or lowered extracellular Ca^2+^ (7, 49). Our experiments show that physiological glucose concentrations (e.g., 8 mM) support sufficient ER Ca^2+^ load (50) to enable CICR bursts triggered by direct pharmacological stimulation of RYR and IP_3_ Ca^2+^ channels. Addition of suprathreshold glucose concentration first activates a prominent IP_3_-dependent transient Ca^2+^ release lasting for several tens of seconds (35). IP_3_ activation is localized to segregated β cell clusters and not coordinated within the whole islet (51). Next, Ca^2+^-dependent mitochondrial metabolism of glucose boosts ATP production (20), that directly decreases the opening probability of K_ATP_ channels (52), and directly activates RYR Ca^2+^ channels (13, 53). High input resistance due to K_ATP_ channel closure enhances the coordination between β cells during the regenerative RYR-CICR activity. We also observed coherent intercellular activity after ryanodine stimulation at substimulatory glucose and this activity was specifically blocked by inhibitory concentrations of ryanodine, suggesting that RYR independently promotes intercellular coordination due to strategic cellular localization of these receptors. The long-term activity of intracellular Ca^2+^ receptors during the plateau phase of β cells must be supported with mobilization of Ca^2+^ from the extracellular space, where VACCs could activate through a large ER Ca^2+^ depletion as has been suggested earlier (49). Our results do not discount a role of plasma membrane K_ATP_ channels and voltage-gated Ca^2+^ entry in β cell function. We speculate that K_ATP_ channels play a critical depolarizing role to support activation of L-type VACCs, reloading the ER Ca^2+^ stores, promoting cell-cell coordination and the long-term regenerative activity, consistent with the human genetics of β cell responsiveness and their clinical utility as targets of sulfonylurea therapy (54). K_ATP_ channels may also play an outsized role in the response to an instantaneous glucose increase from hypoglycemic levels (2–3 mM) to supraphysiological levels (15–25 mM). This is consistent with previous observations suggesting that closure of K_ATP_ channels is not the sole mechanism to depolarize β cells (55). Indeed, other plasma membrane ionic currents have been invoked to explain the complex oscillatory behavior of β cells (2, 30). As an example, multiple Ca^2+^-dependent K^+^ and other K^+^ channel activities have been bundled to explain the elusive, so-called K_slow_ conductance, which could critically shape the typical electrical bursting pattern of β cells after the initial β cell activation (56–59). The details about the physiological glucose responses are even less clear in human β cells and still need to be performed (60).

Glucose plays an essential role in the filling, and therefore also emptying, intracellular Ca^2+^ stores (50). Forced depletion of intracellular Ca^2+^ stores using thapsigargin or extracellular EGTA, increases cytosolic Ca^2+^ concentration, and produces a sustained depolarization, with increased frequency of membrane potential oscillations (7, 49). Slow [Ca^2+^]_c_ oscillations have been shown to persist with only minor modifications when SERCA is blocked with thapsigargin (61, 62) or when SERCA3 has been ablated (63–65). Similarly, reduced activity of the SERCA2 pump to maintain ER Ca^2+^ loading has been shown to disrupt glucose-stimulated calcium signaling (66). Increased frequency of [Ca^2+^]_c_ events was also recorded in our experiments using low extracellular Ca^2+^ concentration. We demonstrate that glucose around the threshold (6–7 mM glucose) supports ER Ca^2+^ release through IP_3_ (35) and RYR release channels. In addition to supporting sufficient Ca^2+^ load in the ER, glucose-dependent effects in β cells provide all key substrates, such as ATP and cAMP, to directly trigger and modulate the activation of intracellular Ca^2+^ release channels, even in the initial absence of plasma membrane depolarization that would increase the opening probability of VACCs. Included in these stimuli is the parasympathetic release of ACh binding to muscarinic ACh receptors (mAChRs) and inducing insulin release via the production of IP_3_ and Ca^2+^ release from the intracellular stores (35, 67–69). Previous studies, including our own, systematically underestimated the role of RYR and IP_3_ receptors due to pre-emptying of intracellular Ca^2+^ stores in too low (0–3 mM) extracellular glucose.

The prominent role for intracellular Ca^2+^ release had strong early support from ^45^Ca^2+^ flux studies (39), but subsequent electrophysiological work challenged this paradigm (49, 53) and evidence accumulated in favor of the dominance of plasma membrane K^+^ channels and VACCs in patterning the dynamics of [Ca^2+^]_c_ events. Even the presence of RYR Ca^2+^ channels in β cells was debated (41, 70). There has been less controversy regarding the expression and roles of IP_3_Rs (71, 72). However, we and others have documented RYR activity and confirmed RYR2 as the most abundant isoform expressed in rat, mouse, and human β cells (18, 19, 21, 73, 74). Indeed, the unique localization and Ca^2+^-release kinetics of each intracellular Ca^2+^ release channel enables the coding of Ca^2+^ signals to control specific cellular (73, 75–77) or intercellular functions. The duality of contributions of both IP_3_ and ryanodine receptors has been previously described to underly complex patterns of intracellular Ca^2+^ regulation of neuronal activity (17). As proposed in our recent theoretical paper, β cell and islet collectives could utilize dual Ca^2+^ sources to adequately respond to a variety of metabolic challenges from those requiring transient hormonal adjustments to those requiring major release of insulin (78). Such a functional arrangement would support both a form of a circuit memory, and risk of a stochastic editing contributing to the pathogenesis of diabetes mellitus (79).

It was proposed that a β cell’s ER is extraordinarily leaky to Ca^2+^ (14). ER Ca^2+^ leak is accelerated by ER stress, leading to β cell apoptosis (19, 21). In addition, excitotoxicity and ER Ca^2+^ overload have been implicated in β cell apoptosis (21) and may also contribute to diabetes pathogenesis. There is strong evidence that ER dysfunction is involved in the pathogenesis of both type 1 and type 2 diabetes (80). In type 1 diabetes, ER dysfunction is a prominent and early feature (81). In type 2 diabetes, Genome-wide association studies have identified multiple RYR2 SNPs with suggestive evidence in dozens of glycemic traits (www.type2diabetesgenetics.org, accessed Feb 2021) (82). Both ER Ca^2+^ load as well as intracellular Ca^2+^ channels can serve as targets for therapy of diabetes mellitus (83).

Pharmacological inhibition of VACCs activity with verapamil was recently reported to have a positive effect on β cell function and survival in adults with recently diagnosed T1D (84), consistent with previous preclinical studies (85). Preclinical studies also demonstrate that inhibition of voltage-gated Na^+^ channels can protect β cells from cytokine-induced death, and reduces diabetes incidence in NOD mice (86, 87). Also, intracellular Ca^2+^ release channels are druggable and were implicated in successful diabetes therapies. For example, RYR2 receptors can be activated by both ATP and PKA (13) and, can therefore be directly stimulated by glucose and modulated by incretins, catecholamines, and peptide hormones. RYRs are a proposed therapeutic target in Wolfram syndrome (88). Abundant physiological data also indicate that CICR from intracellular stores can serve as an “amplifier” of glucose-induced insulin granule exocytosis and plays a central role in incretin-induced insulin secretion (43, 89).

The major strength and limitations of our approach are essentially the same as they have been described for a fresh pancreatic slice preparation, which we introduced in 2001 (26). This is one of the first studies where we could use high spatial and temporal resolution imaging to specifically address intracellular Ca^2+^ receptors. Some of the observed long events may result from phenomena not necessarily connected to insulin secretion, such as movement, cilia protrusion, metabolism, drift of the objective of the microscope. At the moment we do not have a full understanding of the direction and the magnitude of all potential biases beyond the presented experimental evidence. In summary, using powerful new rapid imaging and data analysis methods, we show here that RYR play an important role in glucose stimulated Ca^2+^ signaling in β cells in situ.

## DATA AVAILABILITY

Data will be made available upon reasonable request.

## SUPPLEMENTAL DATA

10.6084/m9.figshare.20279532.v2 Supplemental Material: https://doi.org/10.6084/m9.figshare.20279532.v2.

## GRANTS

M.S.R. received grants by the Austrian Science Fund/Fonds zur Förderung der Wissenschaftlichen Forschung (Bilateral Grants I3562-B27 and I4319-B30). M.S.R., C.E.-M., and J.D.J. received financial support from National Institutes of Health (NIH) R01DK127236. M.S.R., A.S., and D.K. further received financial support from the Slovenian Research Agency (Research Core Funding Program P3-0396 and Projects N3-0048, N3-0133 and J3-9289). J.D.J. received Grants from CIHR and Diabetes Canada.

## DISCLOSURES

No conflicts of interest, financial or otherwise, are declared by the authors. 

## AUTHOR CONTRIBUTIONS

S.S. and M.S.R. conceived and designed research; S.P., J.P., V.P., L.K.B., N.S., M.S.K., and J.D. performed experiments; S.P., S.S., J.P., N.S., D.K., and M.S.R. analyzed data; S.P., S.S., J.P., L.K.B., D.K., A.S., C.E.-M., J.D.J., and M.S.R. interpreted results of experiments; S.P., S.S., J.P., and M.S.R., prepared figures; S.P., S.S., and M.S.R. drafted manuscript; S.P., S.S., J.P., V.P., L.K.B., N.S., M.S.K., J.D., D.K., A.S., C.E.-M., J.D.J., and M.S.R edited and revised manuscript; S.P., S.S., J.P., V.P., L.K.B., N.S., M.S.K., J.D., D.K., A.S., C.E.-M., J.D.J., and M.S.R. approved final version of manuscript.
